# Prediction of functional outcome in bipolar disorder: Effects of cognitive remediation and cognitive psychoeducational group therapy

**DOI:** 10.1192/j.eurpsy.2021.1655

**Published:** 2021-08-13

**Authors:** G. Sachs, A. Erfurth

**Affiliations:** 1st Department Of Psychiatry And Psychotherapeutic Medicine, Klinik Hietzing, Wien, Austria

**Keywords:** bipolar disorder, Functional Outcome, neurocognition, group therapy

## Abstract

**Introduction:**

In bipolar patients cognitive deficits are an important feature. Persisting neurocognitive impairment is associated with low psychosocial functioning.

**Objectives:**

The aim of this presentation is to discuss potential cognitive, clinical and treatment-dependent predictors for functional impairment in bipolar patients.

**Methods:**

In a first study (1) at the Medical University of Vienna 43 remitted bipolar patients and 40 healthy controls were assessed testing specifically attention, memory, verbal fluency and executive functions. In a randomized controlled trial, patients were assigned to two treatment conditions as add-on to state-of-the-art pharmacotherapy: cognitive psychoeducational group therapy over 14 weeks or treatment-as-usual. At 12 months after therapy, functional impairment and severity of symptoms were assessed. In a second, ongoing study, in-patients from a defined catchment area in Vienna (12th, 13th and 23rd district) were assessed via SCIP (Purdon S. 2005. The screen for cognitive impairment in psychiatry: Administration and psychometric properties. Edmonton, Alberta, Canada: PNL Inc.). The SCIP was performed before and after cognitive remediation. The effects of treatment on functioning were measured with the clinical Global Impression Scale (CGI).

**Results:**

Compared to controls, bipolar patients showed lower performance in executive function, sustained attention, verbal learning and verbal fluency. Cognitive psychoeducational group therapy and attention predicted occupational functioning. In the second study, SCIP and CGI values showed improvement after treatment.

**Conclusions:**

Our data support the idea that cognition affects outcome. Bipolar patients benefit from cognitive psychoeducational group therapy in the domain of occupational life. (1) Sachs G et al. Front. Psychiatry, 23 November 2020 | https://doi.org/10.3389/fpsyt.2020.530026

**Disclosure:**

No significant relationships.

